# The impact of digital trade development on China’s export of technology-intensive products: Evidence from importing countries

**DOI:** 10.1371/journal.pone.0321285

**Published:** 2025-04-25

**Authors:** Chenggang Wang, Fan Meng, Tiansen Liu

**Affiliations:** 1 School of Economics and Business Administration, Heilongjiang University, Harbin, Heilongjiang Province, China; 2 School of Economics and Management, Harbin Engineering University, Harbin, Heilongjiang Province, China; Shenzhen University, China

## Abstract

Developing exports of technology-intensive products is a key focus for China’s high-quality foreign trade development. The rapid growth of global digital trade brings new opportunities and challenges for China’s exports of technology-intensive products. This paper utilizes panel data from 2005 to 2022. And we construct an extended gravity model to thoroughly investigate the impact of digital trade development in importing countries on China’s exports of technology-intensive products. The research findings indicate: (1) The development of digital trade in importing countries significantly promotes China’s exports of technology-intensive products. This effect is more pronounced beyond a certain threshold. (2) The reduction of trade costs and increased foreign direct investment play intermediary roles in facilitating the impact of digital trade in importing countries on China’s exports of technology-intensive products. However, institutional distance exerts a negative inhibitory effect on this process. (3) The impact of digital trade development in importing countries on China’s exports of technology products varies due to differences in product types, national income levels, regional characteristics, economic cooperation, and market potential. The conclusions of this paper provide theoretical and empirical evidence for the Chinese government to enhance the efficiency of exporting technology-intensive products.

## 1 Introduction

With the rapid development of the digital economy, China is accelerating the industrialization of digital technologies and the digitization of industries. The Chinese government emphasizes the necessity of developing the digital economy. Concurrently, the government aims to promote deep integration between the digital economy and the real economy. This effort fosters internationally competitive digital industry clusters. Among these efforts, digital trade, an essential component of the digital economy, is increasingly shaping global trade trends. According to the “Report on Digital Trade Development and Cooperation 2023”, global cross-border data flows surged by 120.6% from 2019 to 2022. During the same period, digital services trade also experienced significant expansion, with a growth rate of 36.9%. These growth rates surpass those of global services and goods trade during the same timeframe. Developing digital trade has become a crucial means for countries worldwide to seize new opportunities in the era and address uncertainties in the changing landscape.

Technology-intensive products are those produced using technical means and scientific researchers. These products are characterized by a high degree of mechanization and automation. Technology-intensive products mainly include advanced technology-intensive manufactured goods (aerospace, precision instruments, electronics, etc.), intermediate technology-intensive manufactured goods (machinery, chemicals, etc.), and simple technology-intensive manufactured goods (basic plastic products, furniture, etc.). The level of development in technology-intensive industries can reflect a country’s scientific and technological capabilities. It plays an indispensable role in upgrading a country’s industrial structure and ensuring stable economic development. In recent years, China has strongly supported the development of technology-intensive manufacturing industries. According to data from General administration of customs of the People’s Republic of China, the proportion of technology-intensive manufacturing products in China’s total manufactured goods exports has been increasing annually, reaching 60% in 2023. However, facing a complex international landscape, China’s exports of technology-intensive products encounter issues such as high substitutability and costs [1]. Particularly, uncertainties in the trade policies of some developed countries exert a negative inhibitory effect on the overall export competitiveness of China’s high-tech products [2]. Therefore, how to promote the high-quality development of China’s exports of technology-intensive products has become an urgent issue for academia to address.

As an important global exporter of technology-intensive products, China’s export situation is directly related to industrial development and economic growth. Differences in the level of development of digital trade in importing countries profoundly affect market access, demand structure and competitive dynamics of technology-intensive products in China. Through Internet technology, both sides of the trade have improved the efficiency of transactions, making it easy for technology-intensive products to reach consumers around the world. Moreover, the increase in Internet penetration in importing countries helps to publicize and promote technology-intensive products. This provides strong support for China’s technology-intensive products to develop new space in the international market. Accurately grasping this influence mechanism can provide a powerful basis for Chinese enterprises to formulate scientific and reasonable export strategies, help enterprises accurately docking international market demand, and enhance product competitiveness; at the same time, it can also provide a key reference for the government to formulate forward-looking trade policies, and promote the accelerated upgrading of China’s technology-intensive industries, better integrating into the global digital trade system, and occupying a favorable position in the international economic pattern. This paper utilizes China’s technology-intensive product export data from 2005 to 2022 with 47 countries. Additionally, it incorporates data on the digital trade development levels of importing countries to conduct research. Subsequently, an extended gravity model is constructed. We attempt to explore this issue through a combination of theoretical analysis and empirical testing.

This paper makes the following marginal contributions: (1) It enriches the research on digital trade. Existing studies lack an analysis from the perspective of importing countries on how the development of digital trade promotes China’s exports of technology-intensive products. Moreover, there is limited literature focusing on the transmission mechanisms of this impact. This paper analyzes how digital trade in importing countries affects China’s exports of technology-intensive products. It examines trade costs and FDI as intermediate transmission channels, enriching existing literature and offering new research findings. Therefore, this paper supplements the content on the impact of importing countries’ digital trade development on product exports. (2) This paper extends the application scenario of empirical research methods such as gravity modeling. This paper takes more control variables, including GDP per capita, population size, tariff level, whether it joins the WTO and whether it signs FTA with China, trade and financial freedom. These variables encompass multiple aspects of the economy, trade policy, economic and trade cooperation and economic freedom, thus reducing the interference of other unobserved variables in the results and reducing the error variance. This makes the results more robust and enhances the explanatory power of the model. The validity of the findings is also enhanced. (3) When exploring the impact of digital trade development on exports of technology-intensive products, this paper employs heterogeneity analysis. It conducts heterogeneity analysis on different countries, regions, and types of technology-intensive products, considering each country’s market development and economic cooperation. In this context, the paper deepens the research on the impact of importing countries’ digital trade development on China’s exports of technology-intensive products. Additionally, it inspires new policy directions. When formulating policies related to the export of technology-intensive products, governments need to consider product characteristics and the actual situations in various countries.

The subsequent arrangement of this paper is as follows: (1) Literature review. This section summarizes research on digital trade, exports of technology-intensive products, and the impact of digital trade on exports of technology-intensive products. (2) Theoretical analysis and hypotheses. This section uses economic theory to analyze the underlying logic of how the development of digital trade in importing countries affects China’s exports of technology-intensive products and proposes corresponding hypotheses. (3) Research design. This section includes model construction and data. (4) Empirical analysis. This section comprises model regression, robustness checks, heterogeneity analysis, mechanism testing, and threshold effect testing. (5) Conclusions, implications, and research limitations. This section covers research conclusions, implications, research limitations, and future research directions.

## 2 Literature review

### 2.1 Research on digital trade

The term “digital trade” was first introduced in a report by the United States International Trade Commission in 2013. This report defines digital trade as activities involving the delivery of products and services through the internet. Leaders of various countries issued the “Osaka Declaration on Digital Economy” at the G20 Summit in Osaka in 2019. They emphasized the collective commitment of countries in areas such as data flow and the establishment of international rules for e-commerce in the digital economy. With the diversification of trade forms, the scope and implications of digital trade are gradually expanding. Simultaneously, discussions on the economic effects brought about by the development of digital trade are becoming more profound. The economic impacts of digital trade are manifested as follows: (1) The development of digital trade can enhance a country’s position in the global value chain by optimizing industrial structure [3]. (2) Digital trade positively influences firm competitiveness, primarily through avenues of asset expansion and cost reduction for increased efficiency [4]. (3) Digital trade can promote green consumption, alleviate energy poverty, ultimately enhance environmental quality, and stimulate national economic growth [5]. Some scholars focus on digital trade rules, suggesting that such rules can promote digital service exports by reducing trade costs [6]. Furthermore, high-level digital trade rules can significantly increase the value-added services in international trade. These rules have a more pronounced effect on promoting domestic value-added trade [7]. In recent years, discussions on digital trade policies have increased due to the rapid growth of online transactions. When two countries sign trade agreements containing clauses related to digital trade, their digital trade flows increase. This trend becomes even stronger when the digital trade agreements include more specific rules [8]. In recent years, data security has aroused worries and concerns, and regulation about digital trade has become the focus of countries’ efforts. By comparatively analyzing the trade development strategies and digital trade rules of the United States, the European Union, China, and India, scholars find that different countries have adopted differentiated digital trade policies and data governance approaches. And governments strategically intervene in cross-border data flows to influence the distribution of digital trade gains and increase the share of digital industries [9]. While significant progress has been made in developing legal frameworks, norms, and mechanisms to regulate cyberspace, ongoing challenges remain, including jurisdictional conflicts, regulatory fragmentation, and emerging cyber threats. In addition, ethical and human rights considerations continue to shape the debate surrounding the governance of cyberspace, highlighting the need for a rights-based approach to uphold fundamental freedoms and values in the digital sphere [10].

### 2.2 Research on exports of technology-intensive products

Many scholars have conducted research on exports of technology-intensive products. These studies mainly focus on comparing export competitiveness and influencing factors [11]. By calculating the index of revealed comparative advantage, this research demonstrates that the technological content of China’s exported products has significantly increased. The paper utilizes three indicators from import and export data to support this finding. This is particularly evident in high-tech (electrical and electronic products) and medium-tech (mechanical engineering products) sectors. However, China’s greatest trade advantage still lies in low-tech products [12]. China’s investment in research and development and technological innovation plays a positive role in the export of technology-intensive products for a country [13]. Additionally, population aging can also impact exports of technology-intensive products. However, the relationship between the two is not simply linear. Population aging and the competitive advantage of exports of technology-intensive products exhibit an “inverted U-shaped” relationship. In stages of lower aging, the export competitiveness of technology-intensive products may strengthen with the rapid growth of human capital [14]. Furthermore, command-control environmental regulation negatively correlates with export technological sophistication, while market-incentive and voluntary-participation environmental regulation positively correlate with export technological sophistication [15]. A country’s foreign trade structure, openness, and economic growth are also interrelated. Technology-intensive product exports play a significant driving role in economic development on the basis of openness to foreign trade [16].

### 2.3 Research on the impact of digital trade development on exports of technology-intensive products

Some scholars argue that the development of digital trade in a country has a positive impact on technology-intensive products. Digital trade directly promotes the technological complexity of China’s manufacturing exports and exhibits significant regional heterogeneity. This promotion is most evident in technology-intensive manufacturing industries. Moreover, technological innovation plays a role in reallocating during the process of digital trade. Human capital also influences the technological complexity of manufacturing exports [17]. Additionally, the development of the digital economy has a significant positive impact on the export competitiveness of China’s technology-intensive manufacturing industry. This impact is more pronounced in the eastern regions of China. The integration of manufacturing and the digital economy is higher in these regions compared to the central and western regions [18]. Furthermore, the development of the digital economy significantly enhances the export competitiveness of high-tech manufacturing industries. This impact is minimal on medium to low-tech manufacturing industries. Additionally, due to the relatively high quality of labor in high-tech manufacturing industries. This may trigger a complementary effect between the digital economy and high-skilled labor [19]. Importantly, digital technology applications improve a country’s value chain position by increasing the technological complexity of its exports. And this uplift is more pronounced in technology-intensive and pollution-intensive industries [20]. It is essential to note that the development of digital trade in other countries also influences product export trade. Researchers have found that the development of the digital economy in member countries of the Regional Comprehensive Economic Partnership has a significant impact on technology-intensive products, particularly in low-tech-intensive products. This impact is primarily achieved through reducing bilateral trade costs [21].

### 2.4 Research gap

The academic community has conducted research on the impact of digital trade development on exports of technology-intensive products. However, these studies have certain limitations: (1) In terms of research perspective. Most studies focus on the impact of digital trade development in the exporting country on product exports. They overlook the influence of digital trade development in the importing country. (2) In terms of research methods. The use of gravity models is limited in such studies, and there is a lack of control variable utilization. Importantly, many studies lack the selection of mediating variables. (3) Studies on the impact of digital trade development in importing countries on technology-intensive products lack consideration of country and product heterogeneity. This leads to a certain degree of bias. To address these research gaps, this paper delves into the impact of digital trade development in importing countries on China’s exports of technology-intensive products. We aim to provide valuable insights for enhancing the efficiency of China’s exports of technology-intensive products through this paper.

## 3 Theoretical analysis and hypothesis

### 3.1 Direct impact of importing country’s digital trade on China’s exports of technology-intensive products

Digital trade, as a new form of trade, enhances export trade scale by expanding market boundaries and reducing trade constraints. It greatly expands the market boundaries of international trade [22]. Digital trade centralizes the data of market supply and demand, processes, diffuses, and matches them, facilitating better market matching and more trade transactions. For trade suppliers, the development of digital trade brings many conveniences to technology product producers. Producers can utilize online communication, online transactions, and system matching functions on digital platforms. This can accelerate the circulation of tradable technology products between supply and demand [23]. For trade demand, with the development of digital trade, consumers in a globally interconnected context have access to numerous foreign products. For instance, with the rapid advancements in digital technologies like artificial intelligence, big data, and cloud computing, the demand from importing countries for high-performance chips, advanced software systems, and intelligent devices continues to rise [24]. These new demands provide a broad market space for China’s technology-intensive product exports. Furthermore, advancements in digital technology typically accompany an upgrade in the living standards and consumption structure of residents in importing countries. Residents in importing countries have an increased demand for products with high technological content and superior quality [25]. China’s technology-intensive products continuously improve in quality and performance. They meet the demand of importing country consumers for high-quality products, thereby promoting exports. Moreover, as a new production factor, data profoundly promotes the deep integration of the digital economy and the real economy [26]. It not only participates in various stages of production and circulation. It also generates significant multiplier effects on other production factors, greatly enhancing overall production efficiency [27]. Thus, the vigorous development of digital trade in importing countries plays a positive role in promoting economic growth. It further strengthens residents’ consumption potential, facilitating the expansion of technology product imports. From another perspective, digital trade promotes the digitalized collaborative development of the global industrial chain. It leads to closer cooperation between enterprises in importing countries and China along the industrial chain [28]. China possesses strong production capacity and technological advantages in certain segments of technology-intensive industries. The development of digital trade in importing countries enables better alignment and collaboration along the industrial chain. This joint effort drives the production and innovation of technology-intensive products. Through collaboration, Chinese enterprises not only enhance the quality and performance of technology-intensive products but also reduce research and development costs and risks. This helps strengthen the competitiveness of Chinese technology products in the international market. It creates favorable conditions for exports. Based on this, the following hypothesis is proposed:

Hypothesis 1: The development of digital trade in importing countries has a significant positive impact on China’s exports of technology-intensive products.

### 3.2 Importing country’s digital trade affects China’s exports of technology-intensive products by lowering trade costs

The development of importing country’s digital trade improves the trade environment. The advancement of digital trade makes the transmission of trade information more convenient and efficient, reducing trade costs between China and the importing country. Specifically, trade costs can be divided into two categories, variable trade costs and fixed trade costs. Variable trade costs mainly stem from costs related to transportation, communication, etc., caused by bilateral distances. Fixed trade costs are primarily influenced by non-tariff barriers in multilateral agreements, bilateral agreements, and domestic administrative interventions [29]. In terms of variable trade costs, digital trade, based on internet platforms, provides a more convenient and efficient channel for information exchange. This channel breaks geographical barriers of traditional trade methods, effectively reducing communication and search costs for both sides of the trade. It improves the degree of information asymmetry and enhances trade efficiency [30]. Regarding fixed trade costs, firstly, the development and widespread application of e-commerce platforms help manufacturing enterprises adopt differential competitive strategies. This reduces the entry barriers and market entry costs of the manufacturing industry. Compared to large multinational corporations participating in traditional international trade, more small and medium-sized enterprises benefit from the development of digital trade. They engage in the export of technology products [31]. Secondly, the electronic footprint of importing country consumers can provide a basis for credit scoring [32], including a digital credit assessment system for both buyers and sellers. This helps broaden the scope of credit evaluation and reduce the credit costs of all parties involved. Besides, with the development of digital technologies in importing countries, the degree of informatization of importing country governments will continuously improve [33]. During the trade execution and fulfillment process, relevant institutions of trade parties can effectively reduce the institutional and compliance costs. This is achieved through online connections when exporting technology products. The customs declaration process of digital trade can be completed digitally, significantly saving clearance costs [34]. Therefore, the development of importing country’s digital trade can create favorable conditions for China’s exports of technology-intensive products by lowering trade costs. Hence, the following hypothesis is proposed:

Hypothesis 2: The development of importing country’s digital trade positively impacts China’s exports of technology-intensive products by reducing trade costs.

### 3.3 Impact of importing country’s digital trade on China’s exports of technology-intensive products through FDI promotion

The development of importing country’s digital trade will promote China’s outward foreign direct investment (FDI). In the digital economy era, the level of digital trade development in host countries will become a crucial factor for multinational corporations to consider when investing. Countries with a favorable digital trade development environment, rich digital resources, and well-established digital infrastructure will possess new comparative advantages and be more competitive in attracting foreign investment. Firstly, the construction of digital infrastructure, the operation of digital businesses, and the application of digital devices all require an improvement in the quality of the domestic workforce. Additionally, they necessitate an enhancement in the knowledge system of using digital technologies. Therefore, the development of digital trade provides an incentive for companies to continuously enhance the education level of their employees. This drives the comprehensive improvement of workers’ knowledge literacy and technical skills, thereby enhancing the human capital environment [35]. In today’s world, human capital levels have become a core competitive advantage for a country. Multinational corporations prefer to conduct high-tech research and development in host countries with higher levels of human capital. Therefore, the improvement in the host country’s human capital level can be a favorable factor in attracting foreign investment [36]. Furthermore, the development of importing country’s digital trade implies progress in its digital technology field and innovation capacity. This enhancement also increases companies’ willingness for outward FDI. Technological innovation is a crucial factor in the new round of national competitive advantages. The host country’s technological and innovation levels are important considerations for attracting foreign direct investment. For Chinese companies to achieve mutual benefits, they should focus on the host country’s strategic assets such as innovation and absorption capabilities. The development of digital trade requires innovation as a key production factor to drive the continuous integration of new technologies. This transformation is essential for shifting economic development from being driven by factors like capital. It aims to become innovation-driven [37], making innovation a vital mechanism for high-quality economic development. Companies need to enhance production efficiency through technology. They also need to create business value through more digitized innovative business models [38]. The development of digital trade can enhance innovation efficiency through reducing transaction costs and driving changes in demand. In the development process of digital trade, the favorable innovation environment created by infrastructure construction, policy incentives and talent concentration effects will attract foreign investment. Moreover, the improvement of enterprise innovation and competitiveness can attract foreign investment, which is manifested in the fact that technological innovation will attract upstream and downstream foreign investment in the industry, and model innovation will attract foreign investment in supporting services. Foreign investment will also be attracted by innovation investment opportunities, in some emerging technology industries with high growth potential and innovation vitality, may attract the attention of venture capital institutions. In this context, the development of importing country’s digital trade can improve the investment environment and facilitate foreign investment inflows. Based on this, the following hypothesis is proposed:

Hypothesis 3: The development of importing country’s digital trade positively impacts China’s exports of technology-intensive products through promoting outward foreign direct investment.

### 3.4 Institutional distance plays a negative moderating role

The quality of institutions reflects a country’s level of trade policy, monetary policy, legal system, government governance, etc. It serves as a crucial indicator internationally to evaluate economic institutions. The development of digitization is fundamentally changing the world economic landscape. With the digitization and intelligence of the global value chain, the global layout of multinational companies’ value chains becomes more flexible. The replication and transfer of industrial chains become more convenient [39]. However, with the rise of global trade and investment protectionism, geopolitical risks are continuously increasing. This places higher demands on the institutional quality and environment of importing countries. Additionally, digital trade emphasizes data privacy protection and intellectual property rights protection. These characteristics pose new challenges to current social governance models. The current situation of countries adopting data localization policies to protect privacy adds to these challenges and tests government governance capabilities. The export of Chinese technology products is influenced by differences in rules and perceptions from various countries. This is known as the impact of institutional distance. The unfamiliarity of institutions imposes significant disadvantages on technology product manufacturing companies. The institutional distance between the exporting and importing countries further adds trade uncertainties. Moreover, export companies face more complex operational conditions and higher external communication coordination costs when collaborating with importing country governments and enterprises. This further affects the promotional role of digital trade in technology-intensive products. Specifically, some developed countries attach great importance to the protection of intellectual property rights, and the relevant legal system is perfect and strictly enforced. In contrast, there may be some gaps in China’s IPR protection system in certain aspects, which leads to many problems for Chinese technology manufacturing enterprises when exporting to the United States. For example, some Chinese high-tech enterprises have been investigated and prosecuted by the U.S. side for possible IPR infringement when exporting their technology products to the U.S. in the fields of software and electronic chips. In addition, the European Union’s technical standards and certification system is more complex and strict, such as CE certification in the field of electrical and electronic products. When exporting to the EU, some Chinese manufacturers of electronic and technical products need to spend a lot of time and cost to make their products comply with EU standards and certification requirements. For example, when some Chinese smart home appliance enterprises enter the EU market, they find that the standards of their products in terms of electromagnetic compatibility and other aspects are different from those in China, and they need to redesign and improve their products, which increases the cost of the enterprises and the time for the products to be marketed, and brings uncertainty to trade.

An increase in institutional distance implies differences in the completeness of legal and regulatory systems between trading countries, the level of national corruption, and the expansion of trade barriers [40]. Generally, the greater the institutional distance between trading countries, the more challenging it is to reach consensus on trade cooperation. This adversely affects product imports and exports. Based on this, the following hypothesis is proposed:

Hypothesis 4: Institutional distance negatively moderates the promotion effect of importing country’s digital trade development on China’s exports of technology-intensive products.

**Fig 1:**
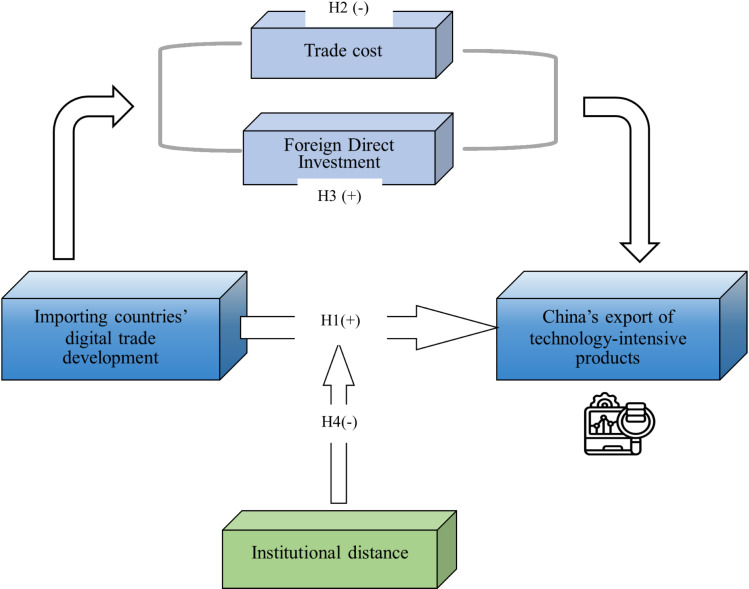
The theoretical model.4 Research design.

In order to strengthen the framework of the theoretical assumptions, we visualize them and the theoretical model of this paper is shown in Fig 1.

### 4.1 Variables

#### 4.1.1 Explanatory variable.

The explanatory variable in this paper is the level of importing country’s digital trade development ($DIGITAL_{jt}$). Currently, there is no clear definition and statistical framework for digital trade internationally. International organizations such as the United Nations Conference on Trade and Development and the World Trade Organization use broad statistical methods. They define digital trade as transactions conducted in digital ordering and digital payment forms, including cross-border e-commerce, digital delivery, and all online transactions and services. This paper adopts a broad definition, referencing existing evaluation systems [41–43]. Ultimately, we select digital infrastructure, digital trade scale, digital innovation environment, and trade potential to measure the level of importing country’s digital trade development (see Table 1).

**Table 1:** Evaluation system for importing country’s digital trade development level.

First-level index	Second-level index	Index attribute	Index weights	Data source
Digital infrastructure	Internet penetration rate (%)	+	0.023	International Telecommunication Union
	Mobile cellular subscriptions (per 100 people)	+	0.014	
	Fixed telephone subscriptions (per 100 people)	–	0.009	
	Fixed broadband subscriptions (per 100 people)	+	0.051	
	Per capita international internet bandwidth (bits/person)	+	0.095	
Digital trade scale	Share of ICT goods exports (%)	+	0.098	World Bank Development Indicators Database
	Share of ICT services exports (%)	+	0.049	
	High-tech products exports (% of total manufactured exports)	+	0.054	
Digital innovation environment	R&D expenditure as a percentage of GDP (%)	+	0.052	
	R&D researchers (per million people)	+	0.072	
	Scientific journal articles	+	0.103	
	Resident patent applications	+	0.203	
Trade potential	Total import and export value (in billion US dollars)	+	0.068	
	Final consumption expenditure (in billion US dollars)	+	0.110	

**Fig 2:**
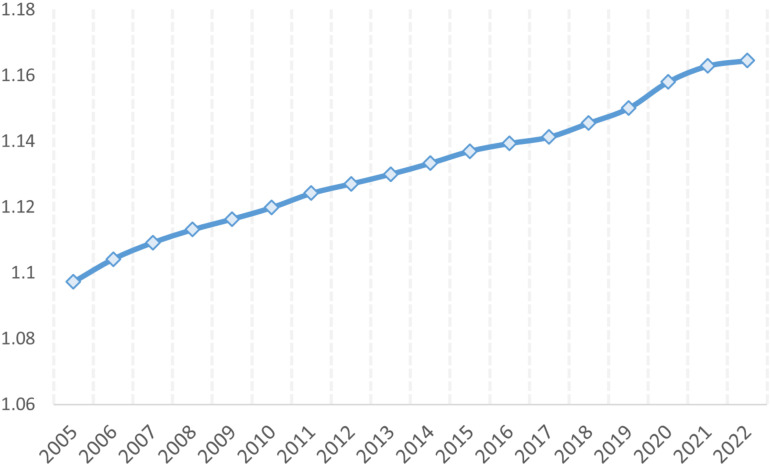
The average level of digital trade development in importing countries.

After selecting the indicators, this paper uses panel entropy method to calculate the level of digital trade development in each country, denoted as $DIGITAL_{jt}$. Fig 2 shows the changing trend of average digital trade development levels among trading nations. In the time dimension, the overall trend of the level of development of digital trade in each country from 2005 to 2022 is upward, from 1.1 to 1.16, an increase of about 0.06. This result shows that countries gradually realize the importance of developing digital trade, and governments have introduced relevant policies focusing on building the development of the digital economy and digital trade-related areas in order to improve the level of national digital trade development. It can be seen from the figure that from 2005–2019, the level of digital trade development has gradually increased from the starting level of about 1.1 to nearly 1.15. The growth in this stage is relatively smooth, reflecting the gradual accumulation and development of the global digital trade in the initial stage. After 2019, the growth of digital trade accelerates further, especially during 2020–2022. This may be related to factors such as the acceleration of global digital transformation, the widespread application of emerging technologies such as big data and artificial intelligence in trade, and changes in the global trade environment. Despite the impact of factors such as the global epidemic during this period, digital trade has maintained its growth trend, showing its strong resilience and adaptability. With the continuous innovation and application of digital technologies, as well as the continued advancement of global digital transformation, global digital trade is expected to continue to grow in the future.

#### 4.1.2 The explained variable.

The dependent variable in this paper is the export value of China’s technology-intensive products ($LnEXP_{ijt}$). Based on data reliability and availability, the total export value of China’s technology-intensive products to each country from 2005 to 2022 is cumulatively ranked. Subsequently, we identify 47 countries with import totals exceeding $500 billion, representing China’s major trading partners. Hence, we select their imports of technology-intensive products as the dependent variable. For the definition of technology-intensive products, this paper relies on previous research [44] and incorporates the International Standard Industrial Classification of All Economic Activities (ISIC), the Harmonized Commodity Description and Coding System (HS), and the classification by the National Science Foundation in the United States. Ultimately, we choose products classified under HS codes in categories six (chemicals and related products), seven (plastics and rubber products), sixteen (machinery, electrical equipment, etc.), seventeen (vehicles, ships, aircraft, etc.), and eighteen (optical, medical instruments, instruments, etc.).

**Fig 3:**
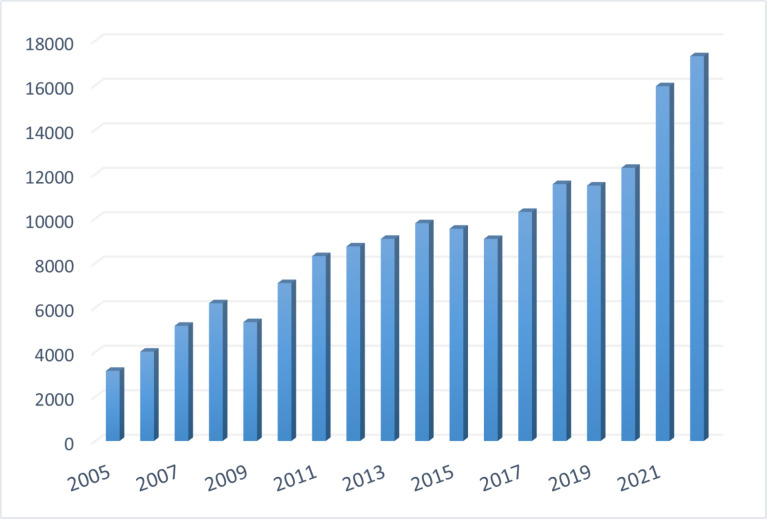
Export trade value of China’s technology-intensive products (in billion US dollars).

From Fig 3, it is evident that the export value of China’s technology-intensive products witnessed a fluctuating increase from 2005 to 2022. The export value of technology products rose from $3151.94 billion in 2005 to $17294.27 billion in 2022, marking a 448.69% increase. Notably, there were significant declines in export value in 2009 and 2016, attributed to the substantial impact of the 2008 financial crisis on China’s high-tech product exports. The comprehensive impact on major export products, markets, and entities served as a direct cause for the decline in high-tech product exports. Additionally, due to insufficient independent innovation in China’s high-tech products, export reliance heavily on processing trade and foreign-invested enterprises is observed [45]. The export dependence on processing trade is contingent on the recovery of international market demand. The uncertainty surrounding the resurgence of demand from major trading partners significantly affects the export of high-tech products through processing trade. In 2016, China also experienced a substantial decline in the total export value of technology products. This decline can be attributed to the impact of falling international commodity prices and exchange rate fluctuations. It leads to a sharp decrease in export trade volume. Furthermore, in the post-financial crisis era, global economic growth slowed down. Some countries, such as those in Latin America, experienced economic downturns, resulting in reduced demand for China’s technology products.

**Fig 4:**
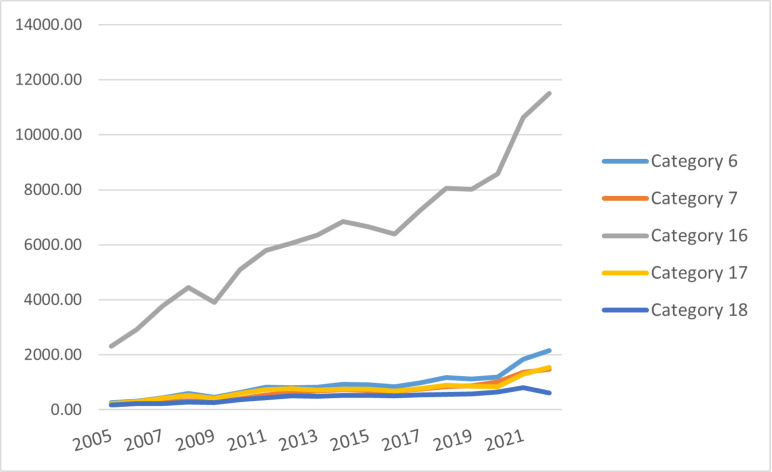
Export trade value of various types of technology-intensive products in China (in billion US dollars).

We conduct an analysis by product type. Fig 4 reveals that from 2005 to 2022, China’s exports of category sixteen technology products lead significantly. This highlights the crucial role of machinery and electrical products in the country’s foreign trade development. Due to their high added value and rapid product turnover and upgrades, machinery and electrical products are prone to generating economies of scale [46]. While the export values of categories six, seven, seventeen, and eighteen technology products show an upward trend, the overall growth rate is relatively modest. This is because for the seventeenth and eighteenth categories of products in the field of these categories of products may be foreign technology leading enterprises to control the core technology, the formation of high technical barriers, foreign enterprises long-term accumulation of patented technologies and R & D advantages, so that the Chinese enterprises are difficult to break through in the short term, restricting the product innovation and upgrading, resulting in slow growth in exports. And in the international market, these products face fierce competition from many enterprises around the world. For example, in the sixth and seventh categories of products, well-known foreign brands have occupied a large market share, and Chinese enterprises are at a disadvantage in terms of brand influence, customer resources, etc., making it difficult to rapidly expand market share and export volume. This export structure reflects China’s high dependence on machinery, electrical equipment, and electronic products, which hinders risk diversification. Moreover, these products have lower technological content and added value compared to instruments. And they limit profit margins for Chinese technology manufacturing export enterprises. Therefore, China needs to optimize its export trade structure to drive economic structural transformation and upgrading.

#### 4.1.3 Mediating variables.

(1) Trade cost (COST)

Following the theoretical analysis in the previous sections, trade costs serve as a crucial channel through which the development of digital trade in importing countries impacts China’s export of technology-intensive products. To address this, the paper employs Novy’s (2013) method to calculate the trade costs of China’s export of technology-intensive products [47]. This results in the intermediary variable COST. The specific calculation formula is as shown in Equation 2:


$$\begin{array}{c} \tau_{ij}={\left(\frac{t_{ij}t_{ji}}{t_{ii}t_{jj}}\right)}^{\frac{1}{2}}-1={\left(\frac{x_{ii}x_{jj}}{x_{ij}x_{ji}}\right)}^{\frac{1}{2\left(\sigma_{k}-1\right)}}-1 \end{array}$$ (1)

Where $\tau_{ij}$ represents the bilateral trade costs between importing country *j* and China *i*, with $t_{ij}$ and $t_{ji}$ denoting the trade costs from China to the importing country and vice versa, while $t_{ii}$ and $t_{jj}$ indicate the domestic trade costs within China and the importing country. $x_{ii}$ and $x_{jj}$ refer to the domestic trade within the manufacturing sectors of China and the importing country during period *t*, while $x_{ij}$ and $x_{ji}$ respectively represent the export values of manufacturing products from China to the importing country and vice versa. The parameter *σ*, denoting the substitution elasticity, is set at 8 following Novy (2013) methodology [47].

(2) Foreign Direct Investment (FDI)

Optimizing the digital trade environment in importing countries enhances investor confidence, attracting more foreign direct investment. Therefore, this paper utilizes China’s FDI flows, commonly used in empirical research, as one of the intermediary variables [48].

(3) Institutional distance ($ID_{jt}$)

In this research, institutional distance is selected as the moderating variable to investigate the impact of digital trade in importing countries on China’s export of technology-intensive products. Drawing on data from six dimensions of the Global Governance Index, the paper calculates the institutional distance between China and the importing country. The distance measurement method proposed by Kogut and Singh (1988) is utilized for this calculation [49]. The specific formula is as follows:


$$\begin{array}{c} ID_{jt}=\frac{1}{n}\overset{n}{\underset{k=1}{\sum }}\left[\frac{{\left(I_{kj}-I_{kc}\right)}^{2}}{V_{k}}\right] \end{array}$$ (2)

Where $ID_{jt}$ represents the institutional distance between China and the importing country, *n* is the number of indicators, $I_{kj}$ and $I_{kc}$ are the scores of the importing country and China on each indicator dimension, and $V_{k}$ is the variance of all countries on the $k^{th}$ dimension. The Global Governance Index primarily assesses six different dimensions: degree of democratic governance, political stability, government efficiency, regulatory efficiency, legal environment, and corruption control.

#### 4.1.4 Control variables.

(1) Per capita GDP of importing and exporting countries ($LnPGDP_{ijt}$): Per capita GDP can represent a country’s level of economic development, serving as the foundation for its trade activities. It is commonly believed that the economic development of an importing country leads to higher consumer spending. This increased purchasing power, in turn, boosts imports. Similarly, economic progress in an exporting country raises consumer spending, increases demand, and may hinder exports.
(2) Population size of importing and exporting countries ($LnPOP_{ijt}$): The growth of China’s population generates more domestic demand, potentially restraining the scale of exports. Population growth in importing countries benefits the market expansion of China’s technology-intensive products and boosts exports.
(3) Level of tariff barriers imposed by importing countries ($MAT_{jt}$): This is represented by the weighted average tariffs levied by importing countries on manufactured goods. Trade barriers set by a country hinder the importation of products. The higher the tariffs on manufactured goods in importing countries, the more they impede the export of China’s technology-intensive products.
(4) Economic and trade cooperation status of importing countries ($WTO_{jt}$ and $FTA_{jt}$): Importing countries joining the WTO and signing free trade agreements with China facilitate resource exchange between nations, expand markets, and enhance economic benefits.
(5) Level of economic freedom in importing countries ($TF_{jt}$ and $FF_{jt}$): Increased trade freedom in importing countries reduces barriers to foreign trade. This prompts Chinese enterprises to enhance innovation and product quality, thus benefiting China’s product exports. A higher level of financial freedom in a country indicates a better financial environment, greater ease of capital flow, and fewer restrictions on foreign investment. Therefore, when China engages in infrastructure development in importing countries, it is easier to obtain financial support. This leads to increased imports and enhanced trade efficiency.

The main variables are defined as shown in Table 2.

**Table 2:** Main variable description.

Type of variables	Variable	Variable declaration	References
Explained variable	$LnEXP_{ijt}$	The natural logarithm of China’s exports of technology-intensive products to importing countries	Chiaroni et al. (2008) [44]
Explanatory variable	$DIGITAL_{jt}$	calculated using the entropy weight method	Li et al. (2024) [41]; Hu et al. (2022) [42]; Zhang (2021) [41]
Mediating variable	COST	${\left(\frac{x_{ii}x_{jj}}{x_{ij}x_{ji}}\right)}^{\frac{1}{2\left(\sigma_{k}-1\right)}}-1$	Novy (2013) [47]
	FDI	China’s direct investment flow to importing countries	Liu and Qian (2021) [48]
Moderating variable	$ID_{jt}$	$\frac{1}{n}\overset{n}{\underset{k=1}{\sum }}\left[\frac{{\left(I_{kj}-I_{kc}\right)}^{2}}{V_{k}}\right]$	Kogut and Singh (1988) [49]
Control variable	$LnPGDP_{ijt}$	The logarithm of the ratio of GDP to total population for both importing countries and China	Tinbergen (1962) [50]
	$LnPOP_{ijt}$	The logarithm of the total population for both importing countries and China	
	$MAT_{jt}$	The weighted average tariffs imposed by importing countries on manufactured goods	Yotov (2022) [51]
	$WTO_{jt}$	Whether the importing country is a member of the WTO	
	$FTA_{jt}$	Whether the importing country has signed a free trade agreement with China	
	$TF_{jt}$	The trade freedom level of the importing country	Guedidi et al. (2024) [52]
	$FF_{jt}$	The financial freedom level of the importing country	

### 4.2 Model construction

The trade gravity model originated from Newton’s law of universal gravitation. Scholars later introduced it into the field of trade to analyze bilateral trade flows and their influencing factors [53]. The original form of the trade gravity model is:


$$\begin{array}{c} T_{ij}=\alpha \frac{Y_{i}E_{j}}{D_{ij}} \end{array}$$ (3)

Here, $T_{ij}$ represents the trade volume from country *i* to country *j*. $Y_{i}$ denotes the output of the exporting country. $E_{j}$ represents the expenditure of the importing country, and $D_{ij}$ signifies the distance between the two countries. Thus, this formula indicates that the trade volume between two countries is directly proportional to the economic size of the two countries. It is inversely proportional to the distance between them [50].

Given that in the context of digital trade, trade in technology products often occurs online, transcending spatial and temporal constraints. Geographical distance is not utilized as an explanatory variable in this paper. In addition to retaining traditional explanatory variables, this paper incorporates factors such as the trade policies of importing countries. It also considers the economic freedom levels in its analysis. Furthermore, to control for resistance factors in importing countries, individual and time fixed effects are included in the extended gravity model construction. This approach also accounts for other country-level characteristics that change over time, such as exchange rate levels and technological developments:


$$\begin{array}{c} LnEXP_{ijt}=\alpha_{0}+\alpha_{1}DIGITAL_{jt}+\alpha_{2}LnPGDP_{ijt}+\alpha_{3}LnPOP_{ijt}+\alpha_{4}MAT_{jt}\\ +\alpha_{5}WTO_{jt}+\alpha_{6}FTA_{jt}+\alpha_{7}TF_{jt}+\alpha_{8}FF_{jt}+\lambda_{j}+\eta_{t}+\varepsilon_{jt}\# \end{array}$$ (4)

In the context where *i* represents China and *j* represents the importing country of technology-intensive products, and *t*denotes the year. $EXP_{ijt}$ represents the total amount of technology-intensive products exported by China *i* to country *j* in year *t*.$DIGITAL_{jt}$ denotes the level of digital trade development score of country *j* in year *t*, where a higher score indicates a more favorable digital trade environment. $PGDP_{ijt}$ signifies the per capita Gross Domestic Product (GDP) of China *i* and country *j* in year *t*. $POP_{ijt}$ represents the population size of China *i* and country *j* in year *t*. $MAT_{jt}$ indicates the weighted average tariffs imposed on manufactured goods by country *j* in year *t*. $WTO_{jt}$ and $FTA_{jt}$ indicate whether country *j* has joined the World Trade Organization and whether it has an effective free trade agreement with China in year *t*. $TF_{jt}$ and $FF_{jt}$ respectively represent the trade and financial freedom levels of country *j* in year *t*. $\lambda_{j}$ is used to control for inter-country differences, $\eta_{t}$ is used to control for time-varying effects, and $\varepsilon_{ijt}$ is the random disturbance term.

### 4.3 Description of the data source

This paper sets the sample time period from 2005 to 2022, involving a total of 47 countries. Data on China’s exports of technology-intensive products ($LnEXP_{ijt}$) are sourced from the United Nations trade database. The calculation data for the importing countries’ digital trade development level ($DIGITAL_{jt}$) are from the International Telecommunication Union and the World Bank databases. Trade cost (COST) data in the mediating variables are from the ESCAP-World Bank database. Foreign Direct Investment (FDI) data are from the Ministry of Commerce of the People’s Republic of China and the National Bureau of Statistics of China. Institutional distance ($ID_{jt}$) data are from the World Governance Index database. Control variables such as the per capita GDP of importing and exporting countries ($LnPGDP_{ijt}$), population size ($LnPOP_{ijt}$), and weighted average tariffs on manufactured goods by importing countries ($MAT_{jt}$) are from the World Bank database. Data on whether the importing country is a member of the WTO ($WTO_{jt}$) are from the official website of the World Trade Organization. Data on whether the importing country has a free trade agreement with China ($FTA_{jt}$) are from the Ministry of Commerce of the People’s Republic of China. Data on the trade freedom ($TF_{jt}$) and financial freedom ($FF_{jt}$) of importing countries are from the Heritage Foundation.

The specific descriptive statistical results are shown in Table 3. On average, there is a significant disparity in the digital trade development levels among countries, with a maximum value of 0.6, a minimum value of only 0.0144, and a standard deviation of 0.0962. It can be observed that the maximum value is approximately 42 times the minimum value. And the concentration trend of digital trade development levels across importing countries is around 0.132. This indicates a pronounced “digital divide” issue. The differences in information infrastructure and the level of digitization of public services vary significantly among nations. This issue exacerbates global trade imbalances.

**Table 3:** Descriptive Statistics of Variables.

Variable	Sample size	Mean	Standard deviation	Minimum	Maximum
$LnEXP_{ijt}$	846	22.94	1.168	19.47	26.56
$DIGITAL_{jt}$	846	0.132	0.0962	0.0144	0.600
$LnPGDP_{it}$	846	8.704	0.587	7.469	9.451
$LnPGDP_{jt}$	846	9.393	1.256	5.526	11.32
$LnPOP_{it}$	846	21.03	0.0271	20.99	21.07
$LnPOP_{jt}$	846	17.58	1.227	15.01	21.07
$MAT_{jt}$	846	4.293	3.814	0	30.1
$WTO_{jt}$	846	0.914	0.281	0	1
$FTA_{jt}$	846	0.183	0.387	0	1
$TF_{jt}$	846	77.22	10.93	0	95
$FF_{jt}$	846	57.12	19.72	10	90

Source: The authors generated this method by using Stata 17

## 5 Results

### 5.1 Baseline regression analysis

This paper conducts random effects, fixed effects, and mixed effects regressions using a gravity model. The F-test indicates that fixed effects are superior to mixed effects, while the LM test shows that random effects are superior to mixed effects, thus excluding mixed effects. The Hausman test statistic is 22.96 with a p-value of 0.0109. It suggests rejection of the null hypothesis that there are no systematic differences between random effects and fixed effects. Therefore, the paper ultimately selects the fixed effects model for regression.

**Fig 5:**
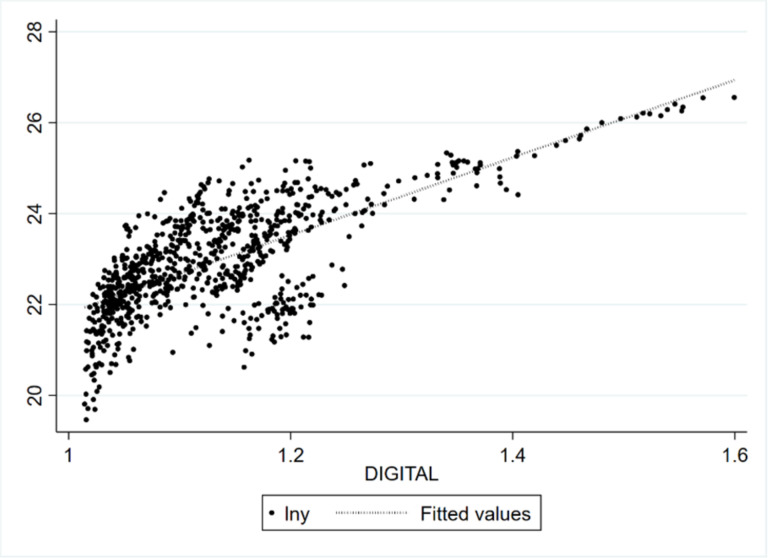
Linear fit plot of importing country’s digital trade and China’s exports of technology-intensive products.

To visually depict the relationship between the importing country’s digital trade and China’s exports of technology-intensive products, this paper creates a linear fit plot of the two variables. Fig 5 illustrates a positive fit relationship. The impact of the importing country’s digital trade on China’s exports of technology-intensive products can be quantified through empirical regression results.

To analyze the relationships between variables in detail, this paper progressively includes various variables in the regression analysis. The baseline regression results are listed in Table 4. In Model 1, the impact of control variables is analyzed, while Models 2 and 3 examine the effects of digital trade. The results indicate that an increase in the importing country’s digital trade development level significantly boosts China’s exports of technology-intensive products. This underscores the crucial role of digital trade as a key trend in international trade, enhancing trade efficiency, expanding trade partners, and diversifying trade formats. China’s exports of technology-intensive products have seen robust growth through internet platforms. Therefore, hypothesis H1 is supported.

**Table 4:** Baseline regression results.

Variable	Model 1	Model 2	Model 3
$DIGITAL_{jt}$		4.231^***^ (0.625)	2.160^***^ (0.528)
$LnPGDP_{it}$	5.910 (3.903)		5.672 (3.865)
$LnPGDP_{jt}$	0.712^***^ (0.047)		0.687^***^ (0.047)
$LnPOP_{it}$	-130.149 (96.491)		-125.700 (95.534)
$LnPOP_{jt}$	1.056^***^ (0.161)		1.041^***^ (0.160)
$MAT_{jt}$	-0.023^***^ (0.006)		-0.022^***^ (0.006)
$WTO_{jt}$	0.068 (0.070)		0.045 (0.069)
$FTA_{jt}$	0.010 (0.040)		0.001 (0.039)
$TF_{jt}$	0.003^*^ (0.002)		0.003^*^ (0.002)
$FF_{jt}$	-0.003** (0.001)		-0.003** (0.001)
Constant term	2676.316 (1995.539)	21.299^***^ (0.071)	2623.879 (1975.644)
Country fixed effects	Yes	Yes	Yes
Time fixed effects	Yes	Yes	Yes
N	846	846	846
R^2^	0.967	0.815	0.969

Note:

*** represents p < 0.01, ***represents p < 0.05,

* represents p < 0.1

Looking at the control variables, the importing country’s per capita income and population size show significant positive effects at the 1% level. This suggests a substantial improvement in productivity accompanying the economic development and population growth of the importing country. Industrialization has notably increased the demand for technology-intensive products, thereby positively stimulating China’s exports of technology products [54]. However, the coefficients for China’s per capita output and total population are not significant. This could be due to inadequate investment and accumulation in basic research in China. Its constraint affects the development of technology-intensive industries and, consequently, the export of related products. The level of tariffs on manufactured goods imposed by the importing country significantly hampers China’ s exports of technology-intensive products. This creates trade barriers for China’s exports. The coefficients for whether the host country is a member of the WTO and whether it has a free trade agreement with China are positive but not significant. This reflects a diminishing institutional dividend in promoting product trade development through joining economic and trade organizations and signing free trade agreements [55]. The impact coefficients of the importing country’s trade freedom and financial freedom on China’s exports of technology-intensive products are significantly positive and negative, respectively. This result demonstrates that an open economic environment in the destination country facilitates the removal of trade barriers for China’s exports of technology-intensive products. It also promotes trade exchanges. Additionally, high financial freedom implies reduced currency interventions. It also suggests increased openness in the financial sector, potentially heightening economic crisis risks. Concerns about uncertainty from both sides could increase trade barriers.

### 5.2 Robustness tests

Robustness tests in this paper involve adjusting the sample period, changing the measurement method of core explanatory variables, removing outliers, and conducting endogeneity tests: (1) Adjusting the sample period. Considering the severe impact of the 2008 global financial crisis on China’s exports of technology products, the sample period is divided into 2005–2008 and 2009–2022 for regression estimation to control for this event’s influence. (2) Changing the measurement method of digital trade development level. Principal component analysis is used instead of entropy weighting to recalculate and regress. (3) Trimming the regression. Due to significant differences in digital trade development levels among countries, the lowest 1% and highest 99% observations in the distribution of core explanatory variables are trimmed before estimation. This is done to prevent extreme values from disturbing the accuracy of the baseline regression results. (4) Endogeneity test. A country’s digital trade development increases product imports. Simultaneously, a country’s frequent trade interactions may serve as a driving force for infrastructure development and enhancing digital trade development. This reciprocal causality leads to endogeneity issues. To address this, following Xin Fan (2020), the lagged one period of the importing country’s digital trade development level is included as an explanatory variable in the regression model to mitigate the impact of reverse causality [56]. The results of these robustness tests are presented in Table 5 (1)-(5). The estimated coefficients of the importing country’s digital trade are all significantly positive at the 1% level, consistent with the baseline model, indicating the robustness of the conclusions drawn earlier.

**Table 5:** Robustness tests.

Variable	(1)	(2)	(3)	(4)	(5)
	2005-2008	2009-2022	Principal component analysis	Trimming variables	Endogeneity test
$DIGITAL_{jt}$	0.815^***^ (0.117)	3.110^***^ (0.627)	0.2840^***^ (0.042)	2.598^***^ (0.545)	2.117^***^ (0.547)
Control variables	Yes	Yes	Yes	Yes	Yes
Country fixed effects	Yes	Yes	Yes	Yes	Yes
Time fixed effects	Yes	Yes	Yes	Yes	Yes
N	188	658	846	846	799
R^2^	0.989	0.973	0.969	0.968	0.968

Note:

*** represents p < 0.01,

*** represents p < 0.05,

* represents p < 0.1.

### 5.3 Heterogeneity analysis

#### 5.3.1 Product heterogeneity.

Considering the diverse nature of different technology-intensive products, their responses to the level of digital trade development may vary. To prevent biased estimation results, following previous scholars’ work [57], this paper categorizes HS2-coded products into low-tech and high-tech categories based on technological complexity. Furthermore, we conduct group regressions, with results presented in Table 6. It is evident that the importing country’s digital trade development significantly promotes high-tech products. This promotion is significant at least at the 1% level, but the impact on low-tech products is not significant. This could be attributed to advancements in digital technology providing impetus for the importing country’s economic development. It leads to changes in market demand structures and increased imports of high-tech products [58]. Moreover, for countries with lower technological levels, the motivation for importing high-tech products stems from a desire to learn and imitate. As a country achieves technological breakthroughs, domestic consumers shift from passive recipients of high-tech products to active choosers. Consumers begin to seek differentiated high-tech products, expanding the demand for high-tech imports. Concurrently, the number of high-tech enterprises and innovation entities domestically increases, fostering the export and import of high-tech products with countries at similar technological levels. The development of the digital economy also accumulates human capital, prompting a shift from importing labor-intensive products to capital-intensive products. This transition in trade structure favors cross-border transactions of high-tech products. Thus, the importing country’s digital trade development increases imports of high-tech products while reducing imports of low-tech products. Notably, the significant coefficient for high-tech products indicates that an increase in the importing country’s digital trade development significantly expands China’ s exports of high-tech products. China should seize this opportunity to optimize its export structure of technology products and promote high-quality development in export trade.

**Table 6:** Heterogeneity analysis of different types of technology-intensive products.

Variable	Low-tech products	High-tech products
$DIGITAL_{jt}$	0.500 (0.584)	2.338^***^ (0.546)
Control variables	Yes	Yes
Country fixed effects	Yes	Yes
Time fixed effects	Yes	Yes
N	846	846
R^2^	0.964	0.969

Note:

*** represents p < 0.01, ***represents p < 0.05, *represents p < 0.1

#### 5.3.2 National heterogeneity.

Considering the economic disparities among countries and regions, differences in education levels and infrastructure exist. Regions with better resources leverage them to further expand their advantages. However, impoverished regions face increasing gaps with advantaged regions due to barriers in technology and information access. A new global wealth gap, the widening “digital divide” is emerging. As there are no low-income countries in the sample countries, this paper categorizes importing countries into high and middle-income countries (based on the latest 2023 World Bank standards). Next, we examine the varying impact of digital trade development on the export of technology products from countries with different income levels. This approach tests whether there is heterogeneity in the positive trade effects of digital trade on technology product exports among countries with different income levels and whether a digital divide exists. Regression results in Table 7 show a significant positive correlation between digital trade development in middle-income and high-income countries and China’s exports of technology-intensive products. Moreover, the impact coefficient for middle-income countries is significantly larger than that for high-income countries, indicating the existence of the “digital divide”. High-income countries have already widely adopted information technology, while many middle-income countries are still in the process of catching up or just beginning. The digital economy can play a significant role in trade processes that have not yet been fully realized. So, it leads to a greater trade promotion effect of digital trade development on middle-income countries.

**Table 7:** Heterogeneity analysis of national income levels.

Variable	High-income countries	Middle-income countries
$DIGITAL_{jt}$	3.434^***^ (0.715)	4.991^***^ (0.813)
Control variables	Yes	Yes
Country fixed effects	Yes	Yes
Time fixed effects	Yes	Yes
N	432	414
R^2^	0.970	0.970

Note:

*** represents p < 0.01, ***represents p < 0.05, *represents p < 0.1.

#### 5.3.3 Regional heterogeneity.

When countries engage in trade, the choice of export destinations is influenced by geographical locations. The role of digital technology in this process may exhibit heterogeneity across different geographic regions. Table 8 reports on the impact of importing countries’ digital trade with China on the export of technology products based on different regional criteria. Countries bordering China and closer Asian countries do not significantly offset technology product exports through the development of digital trade. One possible reason is that with a fixed export volume, in the case of underdeveloped digital trade, a larger proportion of exports goes to countries closer in proximity. As digital trade advances, more opportunities become available, leading to a shift of more trade volume towards countries further away. For distant European, American, and African countries, progress in digital trade by importing countries significantly promotes China’s export of technology products, with larger impact coefficients. This demonstrates that the development of digital trade is more beneficial for distant countries importing from China. The advancement of digital trade transcends spatial and temporal constraints, enhancing resource allocation efficiency.

**Table 8:** Regional heterogeneity.

Variable	Whether the importing country borders China		Geographical location			
	Yes	No	Asia	Europe	Africa	America
$DIGITAL_{jt}$	4.154^***^ (1.084)	1.320^***^ (0.614)	1.365^**^ (0.619)	14.358^***^ (1.378)	17.013^*^ (8.543)	8.049^***^ (1.713)
Control variables	Yes	Yes	Yes	Yes	Yes	Yes
Country fixed effects	Yes	Yes	Yes	Yes	YES	Yes
Time fixed effects	Yes	Yes	Yes	Yes	YES	Yes
N	126	720	342	252	72	162
R^2^	0.986	0.966	0.979	0.961	0.943	0.984

Note:

*** represents p < 0.01,

** represents p < 0.05,

* represents p < 0.1

#### 5.3.4 Heterogeneity analysis of economic cooperation organizations.

Joining an economic cooperation organization benefits by reducing trade barriers among member countries and expanding the sales scope of products. Moreover, increasing economic cooperation enhances a country’s international economic status and investment attractiveness. This promotion leads to trade and economic development. The results of the heterogeneity analysis of economic cooperation organizations, as shown in Table 9, indicate that regardless of whether a sample country is a member of APEC, digital trade promotes China’s export of technology products to varying degrees. When divided based on membership in RCEP, the development of digital trade significantly expands the import scale of technology-intensive products from China by member countries. Therefore, joining economic cooperation organizations can strengthen the trade promotion effects of digital trade.

**Table 9:** Heterogeneity of economic cooperation organizations.

Variable	Whether the importing country joins APEC		Whether the importing country joins RCEP	
	Yes	No	Yes	No
$DIGITAL_{jt}$	1.671^***^ (0.580)	7.690^***^ (1.046)	3.732^***^ (1.378)	0.624 (0.730)
Control variables	Yes	Yes	Yes	Yes
Country fixed effects	Yes	Yes	Yes	Yes
Time fixed effects	Yes	Yes	Yes	Yes
N	270	576	180	666
R^2^	0.981	0.954	0.982	0.966

Note:

*** represents p < 0.01, ***represents p < 0.05, * represents p < 0.1

#### 5.3.5 Market potential.

Based on the global GDP growth rate in 2022, countries are divided into two groups: those with high market potential and those with low market potential for regression analysis [59]. Table 10 reveals that the positive impact of digital trade on China’s export of technology-intensive products is more significant in countries with high market potential. While there is a positive impact in countries with low market potential, it is not statistically significant. This is because countries with high market potential have a higher demand for technology products, attracting a larger proportion of China’s exports of technology-intensive products. In contrast, countries with low market potential are influenced to a lesser extent in their export of Chinese technology products. This is due to the comprehensive and complex nature of digital trade development. Specifically, such countries tend to have relatively low levels of economic development, low overall incomes of their populations and limited spending power. People in some small economies concentrate their spending more on basic necessities. Their willingness to consume and their purchasing power for relatively high-priced technology-intensive products, such as high-end electronic products and precision instruments, are insufficient. Even if the development of digital trade makes it possible to increase purchasing channels, it will be difficult to raise demand significantly. In addition, their industrial structure may be dominated by traditional industries such as agriculture and simple processing and manufacturing, with less demand for advanced technology and technology-intensive products. For example, in some countries where agricultural exports are the main pillar of the economy, the process of industrial digital transformation is slow, and there is no urgent demand for technology-intensive products used to improve production efficiency and innovate products.

**Table 10:** Heterogeneity of market potential.

Variable	High market potential countries	Low market potential countries
$DIGITAL_{jt}$	2.843^***^ (0.694)	0.913 (0.825)
Control variables	Yes	Yes
Country fixed effects	Yes	Yes
Time fixed effects	Yes	Yes
N	558	288
R^2^	0.966	0.979

Note:

*** represents p < 0.01, ***represents p < 0.05, *represents p < 0.1.

### 5.4 Mechanism examination

#### 5.4.1 Mediation test model.

For the mediation mechanism test, this paper adopts the testing method of Zhonglin Wen et al. (2004) [60] and establishes the following model:


$$\begin{array}{c} LnEXP_{ijt}=\alpha_{0}+\alpha_{1}DIGITAL_{jt}+\overset{n}{\underset{i=1}{\sum }}\alpha_{i}control_{jt}+\lambda_{j}+\eta_{t}+\varepsilon_{jt} \end{array}$$ (5)

$$\begin{array}{c} M_{ijt}=\beta_{0}+\beta_{1}DIGITAL_{jt}+\overset{n}{\underset{i=1}{\sum }}\alpha_{i}control_{jt}+\lambda_{j}+\eta_{t}+\varepsilon_{jt} \end{array}$$ (6)

$$\begin{array}{c} LnEXP_{ijt}=\theta_{0}+\theta_{1}DIGITAL_{jt}+\mu M_{ijt}+\overset{n}{\underset{i=1}{\sum }}\theta_{i}control_{jt}+\lambda_{j}+\eta_{t}+\varepsilon_{jt} \end{array}$$ (7)

Here, mediator variable *M* representing bilateral trade costs of manufacturing products and China’s outward foreign direct investment scale. The remaining variables are the same as in Model (4), and bidirectional fixed effects are also included in the model. The results of the mechanism test are shown in Table 11. The first column displays the regression results of the impact of the level of digital trade development on the mediator variable COST, indicating that digital trade development effectively reduces trade costs. The second column shows that after adding the mediator variable COST to the baseline regression model, the coefficients of $DIGITAL_{jt}$ and COST remain significant at the 1% level. This suggests that advancements in digital trade in importing countries can drive China’s export of technology-intensive products by reducing trade costs. Columns three and four present the regression results of the mediator variable FDI. This suggests that advancements in digital trade in importing countries can drive China’s export of technology-intensive products by reducing trade costs. Columns three and four present the regression results of the mediator variable FDI. It can be observed that the development of digital trade In Importing countries promotes China’s export of technology-intensive products by increasing China’s outward foreign direct investment. Both mediator variables play a partial mediating role, with mediation effects accounting for 18.36% and 67.29% respectively. Therefore, hypotheses H2 and H3 pass the test.

**Table 11:** Mediation analysis.

Variable	(1)	(2)	(3)	(4)
	COST		FDI	
COST		-0.003^***^ (0.001)		
FDI				9.850^**^ (5.020)
$DIGITAL_{jt}$	-132.185^***^ (32.812)	1.762^***^ (0.525)	17.736^***^ (34.825)	2.385^***^ (0.535)
Control variables	Yes	Yes	Yes	Yes
Country fixed effects	Yes	Yes	Yes	Yes
Time fixed effects	Yes	Yes	Yes	Yes
N	846	846	846	846
R^2^	0.850	0.969	0.381	0.970

Note:

*** represents p < 0.01,

** represents p < 0.05, * represents p < 0.1.

These results indicate that the development of digital trade in importing countries reduces the cross-border market information search costs. These costs are caused by institutional and cultural differences among countries. Additionally, it alleviates the cultural gap formed by geographical barriers [61]. It makes it easier for importing country consumers to accept Chinese technology products. Furthermore, the development of digital trade in importing countries enhances the input-output efficiency of capital elements [62]. Resource allocation through international capital flows is optimized, attracting more Chinese FDI. This helps reduce the external uncertainty risks and disadvantages faced by Chinese enterprises as outsiders. Consequently, Chinese enterprises can further expand market share, optimize trade channels, and create favorable conditions for the export of technology products.

#### 5.4.2 Moderating effect test.

The impact of the level of digital trade development in importing countries on China’s export of technology-intensive products is subject to the moderating effect of the institutional distance between trading nations. This impact builds upon the analysis of the influencing mechanisms in the preceding sections. Therefore, this paper establishes a moderating effect model to further investigate the influence of institutional distance on the relationship between digital trade and the export of technology products. The specific setup of the moderating effect model is as follows:


$$\begin{array}{c} LnEXP_{ijt}=\omega_{0}+\omega_{1}DIGITAL_{jt}+\omega_{2}ID_{jt}+\omega_{3}DIGITAL_{jt}\times ID_{jt}+\overset{n}{\underset{i=1}{\sum }}\omega_{i}control_{jt}\\ +\lambda_{j}+\eta_{t}+\varepsilon_{jt} \end{array}$$ (8)

Where $ID_{jt}$ serves as the moderating variable representing the institutional distance between the importing country and China. $DIGITAL_{jt}\times ID_{jt}$ denotes the interaction between the level of digital trade development in the importing country and its institutional distance from China. The remaining variables are the same as in Model (4), with bidirectional fixed effects included in the model. The results of the moderating effect test are presented in Table 12. The first column shows the baseline regression results, with a significant positive regression coefficient for the level of digital trade development in the importing country. The second column displays the regression results after incorporating institutional distance. The coefficient of the interaction term between digital trade development and institutional distance is significantly negative. This indicates that the institutional distance between the importing country and China weakens the positive effect of digital trade development in the importing country on China’s export of technology-intensive products. Hence, hypothesis H4 is supported.

**Table 12:** Moderation effect analysis.

Variable	(1)	(2)
$DIGITAL_{jt}$	2.160^***^ (0.528)	2.282^***^ (0.520)
$ID_{jt}$		-0.001 (0.023)
$DIGITAL_{jt}\times ID_{jt}$		-1.042^***^ (0.193)
Control variables	Yes	Yes
Country fixed effects	Yes	Yes
Time fixed effects	Yes	Yes
N	846	846
R^2^	0.967	0.969

Note:

*** represents p < 0.01, ***represents p < 0.05, * represents p < 0.1.

From the perspective of transaction costs, when the institutional distance between two countries is large, differences in legal and regulatory systems increase contract enforcement costs. Moreover, varying institutional environments make it harder for businesses to obtain market information from each other. For instance, in countries with ambiguous market access rules, Chinese exporters of technology products may struggle to understand local quality standards accurately. They may also face challenges in grasping certification requirements and other detailed information about technology products. This necessitates more time and resources for research and consultation, increasing information gathering costs and subsequently affecting technology product exports. Analyzing from the angle of market adaptability, in countries with strict environmental regulations, there are higher requirements for the energy consumption and environmental friendliness of technology products. If Chinese technology products do not meet these standards, they may struggle to enter the market of that country [63]. Moreover, due to a large institutional distance, Chinese enterprises may find it challenging to quickly adjust products to meet the standards of the other country, hindering technology product exports. From a market competitiveness perspective, in countries with a significant institutional distance from China, local enterprises may gain a competitive advantage due to their familiarity with local regulations. They can better leverage local policy incentives or social relationships. In contrast, Chinese exporters of technology products may face disadvantages in market competition. They may also encounter higher market competition risks, leading to obstacles in technology product exports.

### 5.5 Threshold effect test

In addition to the linear impact effect, the import country’s digital trade on China’s technology-intensive products exhibits a nonlinear impact effect. On one hand, the nature of digital trade itself determines its nonlinear development. As a new form of economic activity, digital trade naturally progresses through stages of emergence, growth, maturity, and decline, showcasing nonlinear characteristics in its development [64]. On the other hand, differences in economic development foundations, geographical locations, levels of education and culture, and resource endowments lead to variations in the recognition and acceptance of digital trade among different countries. This results in a nonlinear development status of digital trade, which in turn affects China’s technology product exports. Therefore, this paper considers the import country’s levels of digital trade development and economic development as threshold variables and constructs the following threshold effect model:


$$\begin{array}{c} LnEXP_{ijt}=\psi_{0}+\psi_{1}DIGITAL_{jt}\times I\left(H_{it}\leq \phi \right)+\psi_{2}DIGITAL_{jt}\times I\left(H_{it}>\phi \right)+\\ \overset{n}{\underset{i=1}{\sum }}\psi_{i}control_{jt}+\lambda_{j}+\eta_{t}+\varepsilon_{jt} \end{array}$$ (9)

Where $H_{it}$ represents the threshold variable, namely the level of digital trade development and economic development in the importing country. *ϕ* denotes the threshold value. $I\left(\cdot \right)$ is the indicator function, taking a value of 1 when the condition inside the parentheses is satisfied, and 0 otherwise. The remaining variables are the same as in Model (4), with bidirectional fixed effects included in the model.

Following the approach of Hansen (1999), this paper initially employs the Bootstrap method with 300 resamples to validate the reasonableness of the threshold model and determine the number of thresholds [65]. Table 13 presents the results of the threshold effect test for the level of digital trade development and economic development in the importing country. The results indicate that the level of digital trade development in the importing country passes the single threshold test at a significance level of 10%. The level of economic development in the importing country passes the single threshold test at a significance level of 1%.

**Table 13:** Threshold effect test results.

Variable	Threshold	F-value	P-value	Threshold estimation value	10% critical value	5% critical value	1% critical value
$DIGITAL_{jt}$	Single threshold	40.89	0.067 *	1.101	37.014	45.561	59.922
	Double threshold	21.56	0.270	1.148	32.067	37.942	51.333
	Triple threshold	14.59	0.593	1.172	35.113	40.922	49.517
$LnPGDP_{jt}$	Single threshold	67.82	0.003***	8.149	36.206	43.559	54.764
	Double threshold	25.55	0.197	8.608	33.876	43.205	64.342
	Triple threshold	21.19	0.383	9.273	34.279	41.732	53.661

Source: Compiled by the authors based on the results from Stata 17

**Fig 6:**
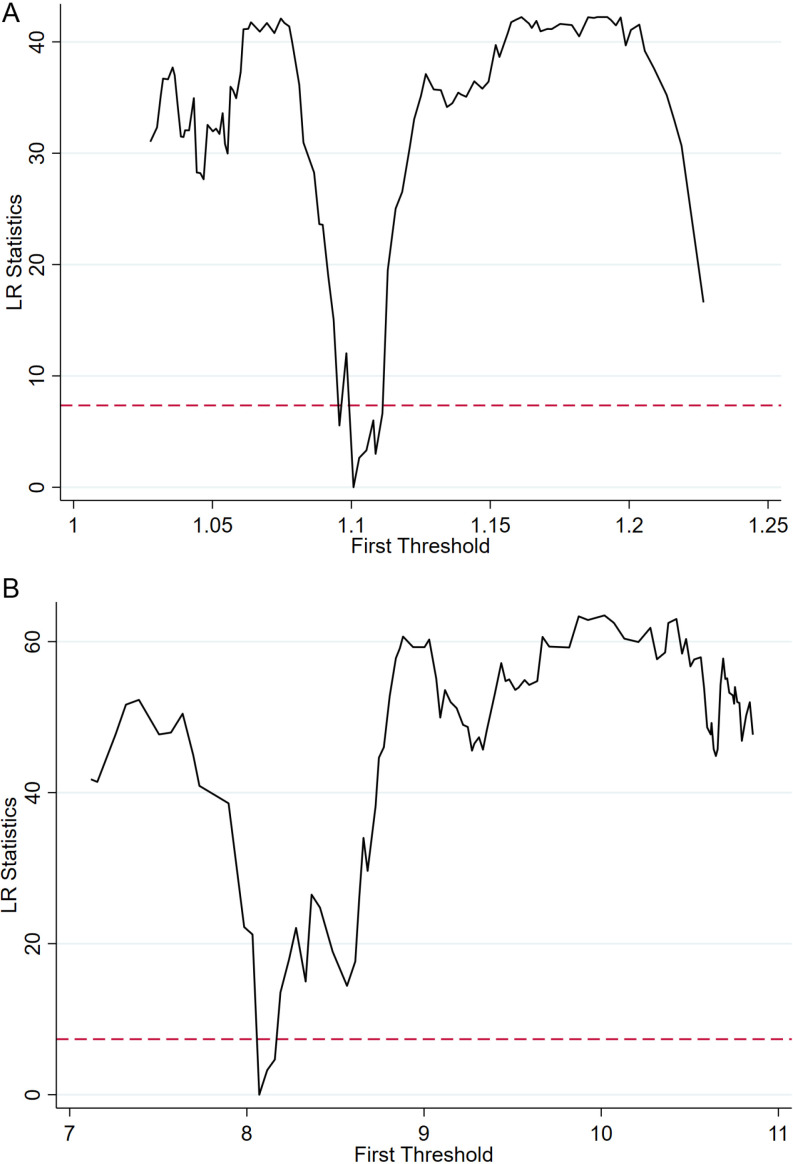
Single threshold likelihood ratio function.

To further verify the reliability of the above threshold values, this paper plots the likelihood ratio functions. As shown in Fig 6, panels (a) and (b) depict the LR plots with the level of digital trade development and economic development in the importing country as threshold variables, respectively. Observations reveal that the threshold values corresponding to the lowest points in both plots fall within the 95% confidence interval (below the dashed line). This indicates that the two-threshold values output by the single threshold effect are indeed valid. Therefore, the impact of the level of digital trade development and economic development in the importing country on China’s technology-intensive product exports exhibits a single threshold nonlinear relationship.

Based on the results of the threshold effect test, a panel threshold regression is conducted on the model. Table 14 presents the regression results of the threshold effect model with the level of digital trade development and economic development in the importing country as threshold variables. In column (1), when the level of digital trade development in the importing country is ≤ 1.101, the coefficient of the impact of digital trade development on China’s technology-intensive product exports is 2.083 at a significance level of 1%. When the level of digital trade development in the importing country is > 1.101, the coefficient increases to 2.305 at a significance level of 1%. In column (2), when the level of economic development in the importing country is ≤ 8.149, the coefficient of the impact of digital trade development on China’s technology-intensive product exports is 3.618 at a significance level of 1%. When the level of economic development in the importing country is > 8.149, the coefficient increases to 4.007 at a significance level of 1%. This indicates a nonlinear impact of digital trade development in the importing country on China’s technology-intensive products. Moreover, when the levels of digital trade development and economic development in the importing country are in higher threshold ranges, the promotion effect of digital trade development on China’s technology product exports is stronger.

**Table 14:** Threshold panel model regression results.

	(1)	(2)
	Importing country’s level of digital trade development single threshold	Importing country’s level of economic development single threshold
$DIGITAL_{jt}\times I$ $\left(DIGITAL_{jt}\leq 1.101\right)$	2.083^***^ (0.539)	
$DIGITAL_{jt}\times I$ $\left(DIGITAL_{jt}>1.101\right)$	2.305^***^ (0.529)	
$DIGITAL_{jt}\times I$ $\left(LnPGDP_{jt}\leq 8.149\right)$		3.618^***^ (0.579)
$DIGITAL_{jt}\times I$ $\left(DIGITAL_{jt}>8.149\right)$		4.007^***^ (0.573)
Control variables	Yes	Yes
Country fixed effects	Yes	Yes
Time fixed effects	Yes	Yes
N	846	846
R^2^	0.698	0.529

Note:

*** represents p < 0.01, ***represents p < 0.05, * represents p < 0.1.

## 6 Conclusions, implications and research limitations

### 6.1 Conclusions

Based on publicly available data from 47 countries, this paper constructs an extended gravity model to regress the impact of the importing country’s level of digital trade development on China’s exports of technology-intensive products. Additionally, robustness checks and heterogeneity analysis are conducted on the regression results. Furthermore, mechanism tests and threshold effect examinations are carried out to explore the transmission mechanisms and conditions of the promotional effect. This paper combines the study of digital trade in importing countries with China’s exports of technology-intensive products, which can incorporate more considerations about digital factors in international trade theory, make the theory more in line with the current digital reality of global trade, and supplement and improve the content of international trade theory about trade influencing factors and trade patterns. At the same time, for the study of the development of digital trade in importing countries on China’s exports of technology-intensive products, we have constructed a systematic theoretical analytical framework, which provides an important theoretical reference for the subsequent related theoretical research and helps other scholars to expand and deepen their theories when studying similar issues. In conclusion, the following findings are obtained:

(1) Overall, over the time span from 2005 to 2022, the level of digital trade development in each importing country shows a gradual increase, with the average level steadily rising from 1.122 to 1.130. This trend signifies the vigorous emergence of the global digital economy. It also reflects a profound transformation of international trade structures towards greater digitalization and intelligence. However, it is worth noting that despite the overall positive development trend, there exists a highly uneven status in the actual development levels of countries in the field of digital trade. In short, the phenomenon of the “digital divide” exists and is quite severe. This conclusion aligns with previous research findings [66].
(2) The development of digital trade in importing countries significantly promotes China’s exports of technology-intensive products. However, the impact of digital trade on different types of technology-intensive product exports exhibits heterogeneity. This conclusion refines the work of earlier scholars [67]. The promotional effect of digital trade on high-tech product exports is more pronounced, while its impact on medium to low-tech product exports is weak. Moreover, the extent of this impact varies depending on differences in national income levels, geographical regions, economic cooperation status, and market potential.
(3) In the process of driving China’s exports of technology-intensive products, the development of digital trade in importing countries plays a crucial mediating role by reducing trade costs and increasing foreign direct investment. The deepening of digital trade in importing countries can significantly lower transaction costs, optimize international trade processes, and enhance trade efficiency. These changes provide a more convenient and efficient channel for China’s technology-intensive products to enter the international market, thereby promoting export growth. However, when there is a significant institutional distance between China and the importing countries, the promotional effect of importing countries’ digital trade development on China’s technology product exports may be somewhat inhibited. This conclusion extends scholars’ research, suggesting that institutional distance may hinder firms’ R&D innovation capabilities, thus impacting export performance negatively [68].
(4) The development of digital trade in importing countries does not exhibit a linear relationship with the promotion of China’s technology-intensive products. Instead, there exists a specific threshold value. When the level of digital trade development and economic development in importing countries has not reached the threshold value, this promotional effect is relatively limited. Despite providing potential market opportunities for Chinese products through their digital trade and economic growth, various constraints still apply. However, once these two indicators surpass this threshold value, the promotional effect on China’s technology-intensive product exports will significantly strengthen. This result also implies that China needs to focus more on target market selection and strategy formulation when promoting exports of technology-intensive products.

### 6.2 Implications

(1) China should strengthen digital trade cooperation with other countries and share the dividends of digital economic development. Firstly, China should further deepen its cooperation with other countries in digital technology innovation, strengthen digital cultural exchanges and borrowing, and jointly promote digital trade and economic cooperation. In terms of talent cultivation, China should support higher education institutions to set up digital trade-related disciplines, strengthen the cultivation of top-notch innovative talents, deepen cooperation between schools and enterprises, government and enterprises, and support enterprises to strengthen the training of professionals, so as to cultivate a batch of digital trade talents who are familiar with the rules of international digital trade, and who have the ability to innovate and have an international vision. Secondly, for countries lagging behind in the development of digital trade, we can appropriately help them carry out digital infrastructure construction. This can not only narrow the “digital divide”, but also help reduce the loss rate of technology-intensive products in the journey and improve trade efficiency. China can provide technical training services to help importing countries improve their technical level and human resources quality, targeting their weaknesses in digital technology and related industrial technology. Through technical assistance programs, it can help importing countries establish relevant technical infrastructure and application systems to create a better market environment for the export of China’s technology-intensive products. Finally, in the face of new trends and problems in the formulation of global digital trade rules, China urgently needs to promote the formation of “China’s rules” in the field of digital trade. China should, on the premise of ensuring national security, seek strategies that take into account the reasonable boundaries of cross-border data flows and promote the diversified development of digital trade. At the same time, it should actively participate in the World Trade Organization, the Group of Twenty, APEC and other bilateral and regional rules on digital trade, make Chinese voices heard, contribute Chinese wisdom, and push for the establishment of a fair, reasonable and inclusive global digital trade rules system.
(2) Chinese enterprises should develop marketing strategies according to local conditions. For different markets and product types, Chinese enterprises should formulate corresponding marketing and promotion programs. Enterprises should study the market demand characteristics and trends of developed countries and countries with high market potential, combine their own technological advantages, define the target customer groups and application scenarios of their products, and develop technology-intensive products that better meet the local market demand. At the same time to improve product quality and performance, the introduction of advanced production equipment and quality management system, strengthen the quality control in the production process to ensure product stability and reliability. Enterprises should also improve the after-sales service system, establish localized after-sales service teams in developed countries and countries with high market potential, and provide timely and efficient technical support and maintenance services. Enterprises should build diversified marketing channels, make full use of social media, online platforms and other digital marketing to improve product brand awareness and market influence. High-tech manufacturing enterprises should continue to increase R&D investment, strengthen technological innovation and product optimization, keep up with technological trends, pay attention to global digital trade-related technological developments, apply new technologies to product development and upgrading in a timely manner, meet the needs of high-end overseas markets, improve the competitiveness of technical products, and promote the optimization of the structure of China’s product exports. The technology manufacturing industry should focus on reducing production costs and environmental impacts, improving the quality and performance of product supply, and enhancing the industry’s competitiveness and sustainable development capability.
(3) China should make good use of digital technology to optimize the trade chain of technology-intensive products. First, the Government should formulate and improve support policies for the export of technology-intensive products, such as export tax rebates, tax incentives and financial subsidies, so as to reduce the operating costs of enterprises. Through the establishment of a special fund, it should support enterprises to carry out digital trade-related technology research and development, innovation and application, and market expansion activities, and encourage them to increase their investment in digital technology, intelligent manufacturing and other fields. Secondly, the Chinese government should guide the reasonable and orderly cross-border layout of the digital trade industry chain, and promote close synergy between the platforms on the consumer side of the sea and the platforms on the industrial side at home. At the same time, China should encourage outbound investment and support the upstream and downstream of the industry chain, such as smart logistics and mobile payment, to go overseas. These initiatives aim to create a number of special digital intellectual industrial belts and cultivate a number of brand projects with Chinese characteristics. Finally, the government wants to improve the efficiency of customs clearance for export goods and empower the enhancement of port informatization and intelligence. At the same time, we need to enhance remote delivery capabilities and deepen China’s connectivity with other countries trading technology-intensive products. By collaboratively regulating and reducing taxes and fees in the export chain, we need to further reduce trade costs and optimize the promotion of cross-border trade facilitation. At the enterprise level, enterprises should strengthen cooperation with local enterprises, establish strategic partnerships with enterprises in importing countries, and share resources and complement each other’s advantages through joint ventures, cooperation, mergers and acquisitions. Cooperation with local enterprises can help China’s enterprises better understand local market information and customer demand, and utilize the sales channels and brand influence of local enterprises to quickly open the market and reduce market development costs. Industry associations should build a timely information service platform for digital trade and foreign investment, collect and organize market information, policies and regulations, and industry dynamics in importing countries to provide enterprises with timely, accurate and comprehensive information support. Through the platform, it releases supply and demand information of technology-intensive products, trade opportunities, investment projects, etc., promotes exchanges and cooperation among enterprises, and helps enterprises reduce information acquisition costs and transaction costs.

### 6.3 Research limitations and future research directions

Due to limitations in our own cognitive level and understanding, this paper still has some shortcomings. These limitations will guide our future research focus:(1) The sample size of this paper is limited. Due to data availability and completeness, this paper selects 47 countries with total imports of technology-intensive products from China exceeding $500 billion. However, this may not cover all of China’s trading partners. A small sample size could affect the accuracy and generalizability of the results. In future research, we will strive to expand the sample size to enhance the persuasiveness of empirical results. (2) The intermediate and moderating variables involved in the model are not comprehensive, simplifying or neglecting the interactions between these variables. This paper only selects trade costs, foreign direct investment, and institutional distance as intermediate and moderating variables, which are relatively few. Potential intermediate variables such as enterprise innovation and customs clearance efficiency are not considered. Subsequent research should consider a more diverse range of intermediate and moderating variables. This approach will lead to a more systematic and comprehensive study of the factors affecting China’s exports of technology-intensive products. (3) The research scope of this paper is relatively narrow. Currently, our analysis is limited to the export dynamics of Chinese technological products and does not comprehensively cover other countries with development potential, such as Brazil, India, and South Africa. The limitation of this research perspective may result in an incomplete and superficial understanding of the global trade patterns of technology products. Therefore, future research should aim to broaden the analytical framework by including more emerging economies to obtain more comprehensive and accurate conclusions.

## Supporting information

S1 Data The data basis for empirical testing. (XLSX)
